# Seasonal dynamics of influenza in Brazil: the latitude effect

**DOI:** 10.1186/s12879-018-3484-z

**Published:** 2018-12-27

**Authors:** Alexandra Almeida, Cláudia Codeço, Paula Luz

**Affiliations:** 1 Escola Nacional de Saúde Pública, FIOCRUZ, Rio de Janeiro, Brazil; 2 Programa de Computação Científica, FIOCRUZ, Rio de Janeiro, Brasil; 3 Instituto Nacional de Infectologia Evandro Chagas, FIOCRUZ, Rio de Janeiro, Brasil

**Keywords:** Influenza, Seasonality, Wavelet decomposition, Circular

## Abstract

**Background:**

Influenza is a global transmissible disease. Its dynamics is far better understood in temperate climates than in the tropics. We aim to close this knowledge gap between tropical and temperate regions by showing how the influenza seasonality evolves in Brazil, a tropical country that encompasses a wide range of latitudes and six climatic sub-types.

**Methods:**

We analyzed a state-level, weekly Syndrome of Acute Respiratory Disease (SARI) incidence data ranging from 2010 to 2016. We combined two techniques hierarchically: first the wavelet decomposition technique to detect annual periodicity and then circular statistics to describe seasonal measures of the periodic states.

**Results:**

We found significant annual periodicity in 44% of the states. For these, we calculated several seasonal measures such as the center of gravity or mean timing of activity. The relationship between the seasonal signatures and latitude was clear and statistically significant. States with seasonal signature are clustered along the coast. Most Amazonian and Central West states exhibit no seasonal behavior. Among the seasonal states, influenza starts in Northeast region, spreading southbound.

**Conclusions:**

Our study advances the comprehension of influenza seasonality in tropical areas and could be used to design more effective prevention and control strategies.

**Electronic supplementary material:**

The online version of this article (10.1186/s12879-018-3484-z) contains supplementary material, which is available to authorized users.

## Background

Influenza is caused by viruses that infect the upper respiratory tract. It is characterized by sudden onset of high fever, myalgia, headache, and severe malaise, non-productive cough, sore throat, and rhinitis [1]. The disease severity ranges from mild illness to severe cases that may evolve to death, particularly among high-risk groups. Though prevention is possible with vaccines updated annually [2], worldwide epidemics are estimated to result in 3 to 5 million severe cases per year, causing 250,000 to 500,000 deaths [3]. Influenza epidemics have been well studied in high-income countries within temperate regions [4–6]. In these regions, the seasons are well marked, resulting in a well behaved disease pattern [7]. In the tropics, seasons are not that well marked resulting in a less predictable seasonal dynamics. There are reports of semi-annual or year-round influenza activity [8, 9]. Studies of influenza in the tropics started recently. We found reports for: Brazil [10, 11], Vietnam [12], Madagascar [13], Myanmar [14] and China [15].

There are also a few ecological studies linking the influenza dynamics to differences in latitude. Some of them associate these differences to different climatic variables [10, 15–22]. These studies use a myriad of methods, sometimes more akin to a data visualization than to statistical analysis. Moreover, *ad hoc* definitions of seasonality are commonly used. However, to assess the complex interactions of influenza with the environment, a thorough understanding of the temporal dynamics and the periodicity of influenza is needed.

Brazil is an interesting case study due to its continental dimension. Its territory ranges from 5 degrees north to 35 degrees south of the Equator line, encompassing six climatic sub-types [23]. The mortality rate by influenza averages 1.01 deaths per 100,000 inhabitants per year [24]. A further advantage of a single country study is that it minimizes issues of differences in the data collection procedures.

Studies on the ecology of influenza and its temporal dynamics in Brazil are recent. It started when Alonso et al. [10] produced a state-level study, using monthly mortality rates for the 1979-2001 period. Within a Fourier analysis framework, they show a relationship between latitude and deaths, both in intensity as in the time location of the peak activity. Later, Mello et al. [11] analyzed influenza isolated activity from two state capitals: São Paulo in the Southeast region and Belém in the North of the country. Using this rather limited sample, they advocate the need to use latitude information into the national vaccination calendar; and Schuck-Paim et al. [25] studied the impact of the 2009 influenza pandemic relative to mortality rates for all Brazilian states and also showed significant latitude differences.

The main goal of this article is to study the dynamics of influenza in Brazil at state-level, to verify where and when it exhibits seasonal behavior and explore its characteristics. We hypothesized that the diversity of environments across states would yield a variety of behaviors and just with dynamic techniques it would be possible to unearth the different seasonal patterns.

We used weekly data on SARI cases (hospitalization and deaths) related to influenza, to assess whether a significant seasonal signature is detected at the state-level, across the diverse Brazilian geographic states/scenarios. We adopted a combination of two statistical methods: (i) the wavelet analysis, a modern, dynamic version of the classical Fourier methodology, used here to detect periodic behavior; and (ii) Circular Statistics, a set of tools designed to model variables known to be periodic.

This merger allowed us to uncover a rich pattern of seasonal behavior, with marked dissimilarities between the subtropical states in the South and the tropical states in the North. Comparing the seasonal aspects of different states from a continental tropical country, encompassing so many different latitudes, will support a better understanding of the interactions between disease and ecology, improving strategies for prevention and control.

## Methods

### Surveillance data

Since 2009, Brazil’s epidemiological influenza surveillance implemented a universal and mandatory notification system for hospitalized cases and deaths diagnosed as Severe Acute Respiratory Infection (SARI). The notifications are made *online* through the Notifiable Diseases Information System (SINAN).

In 2009, the SARI case definition was ascertained to capture both influenza- related pneumonia and influenza-related exacerbations of chronic illnesses such as asthma or heart disease [26]. In 2013, the World Health Organization set the current SARI definition as: “an acute respiratory infection with: (i) history of fever or measured fever above 38 Celsius, (ii) cough, (iii) onset within the last 10 days, and (iv) required hospitalization”.

In this study, we analyzed state-level, weekly incidence of Brazil’s SARI starting at the 1st week of 2010 and ending on the 38th week of 2016. It comprises the 26 states and the federal district (referred here as the 27 states). The state-level incidences were calculated by dividing the number of SARI cases reported by each state by its annual population estimated by the Brazilian Institute of Geography and Statistics (IBGE) [27].

### Time series analysis

The time series analysis carried out in this study uses a hierarchical combination of two methods: wavelet decomposition and circular statistics. The first method is used to detect whether or not a given state exhibits an annual seasonality on its SARI data. The second method, applied only at states where the first one provided an affirmative answer, is used to provide statistics of the detected periodic behavior.

#### The wavelet analysis

In 1822, Joseph Fourier astonished the mathematical community by showing that any periodic function (*f* (*x*) = *f* (*x* + *kT* ); for *k* = ± 1*,* 2*,…*) could be represented as an infinite sum of harmonics. Hence, a pure seasonal behavior, one that repeats itself *ad infinitum*, can also be represented in such way.

This classical technique, as well as ARMA, ARIMA or SARIMA analysis all require that the statistical properties of the time series do not vary through the time. This stringent requirement of stationarity makes all these methods unsuitable for many ecological time series studies [28].

The modern, dynamic version of the Fourier method is called the wavelet decomposition/analysis. Wavelets (small waves) are a set of standard functions such as the Hass or Morlet functions. Unlike their Fourier counterparts, the sine and cosine functions that extend throughout infinity; a wavelet is a compact function, designed to capture local phenomena.

We used wavelet analyses to explore the prevailing periodicity of SARI in all Brazilian states. For each time series, we compute the wavelet power spectrum to verify directly if the data exhibits a significant annual seasonality. The criteria to classify a given time series as seasonal is the following: the average of the *p*-values over the seven-year dataset is below 5%. The null hypothesis is the best fit non-seasonal ARIMA model. The average wavelet power graph illustrates these findings. States that reported more than two years of zero cases were excluded from the seasonality detection.

The references to the wavelets technique are various: For an introduction, see Torrence and Compo [29]. More advanced material can be found on Percival and Walden [30]. For an application of wavelets on ecological studies, see Cazelles et al. [28].

#### Circular statistics

Wherever we can confidently attribute a periodic behavior to a time- series, a new set of tools become available: the techniques referred to as circular statistics have been developed for the analysis of directional/circular data.

The idea is that an angle of 10 degrees is close to the 350-degree angle. Their average, a basic statistical computation, should be the zero angle and not the 180-degree angle; the nonsense result obtained by standard arithmetics. In this study, an event happening in epidemiological weeks 1 and 2 is considered close in time to events in weeks 51 and 52 [31].

This way of looking at the data has its own set of statistical distribution and visualization techniques. The most used distribution for circular data is the von Mises. Its density is given by:

1 $$ f\left(\theta \right)=\frac{1}{2\pi {I}_0\left(\kappa \right)}{e}^{\kappa \kern0.5em \mathit{\cos}\left(\theta -\mu \right)} $$

*θ* is an angle between zero and 2*π*, with a corresponding epidemiological week of *θ* × 52*/*2*π*. *μ* is the location parameter or the sample mean.

*κ* is a positive concentration parameter. As *κ* approaches zero, the distribution becomes the uniform in the circle, meaning year-round activity. As *κ* grows, the action becomes more concentrated in time.

For visualization purposes, a circular variant of the histogram is the rose diagram. It is a circular histogram where the distance from the origin is proportional to the square root of its incidence. This ensures that the area of the sector is proportional to the group frequency [32].

For all annual seasonal states, we performed the Rayleigh’s test of uniformity to verify if the concentration parameter *κ* estimated are different from zero, rejecting the hypothesis of a uniform distribution, favoring a unimodal shape.

In this particular study, we used circular statistics to access measures of SARI activity and temporal concentration for each state: (i) the average yearly incidence, (ii) the center of gravity, interpreted as the epidemiological week that in average has the largest incidence and (iii) the concentration parameter of the fitted von Mises distribution.

Standard references on circular statistics can be found in [33–35].

The statistical software R was used for all statistical analyses (version 3.3.0). The packages WaveletComp (version 1.0) [36] and Circular (version 0.4-93) [37] were used to perform the respective analyses.

## Results

Our database contains weekly SARI incidence time-series from all 26 Brazilian states plus the federal district. We refer to them as the 27 Brazilian states. 21 states are situated in the tropical climate zone, corresponding to 81% of the territory. Towards the South lies the subtropical climate zone, with six states representing 14% of the territory. A smaller region is the semi-arid zone, occupying the other 5% of the territory. No state capital lies in it [23].

**Fig. 1:**
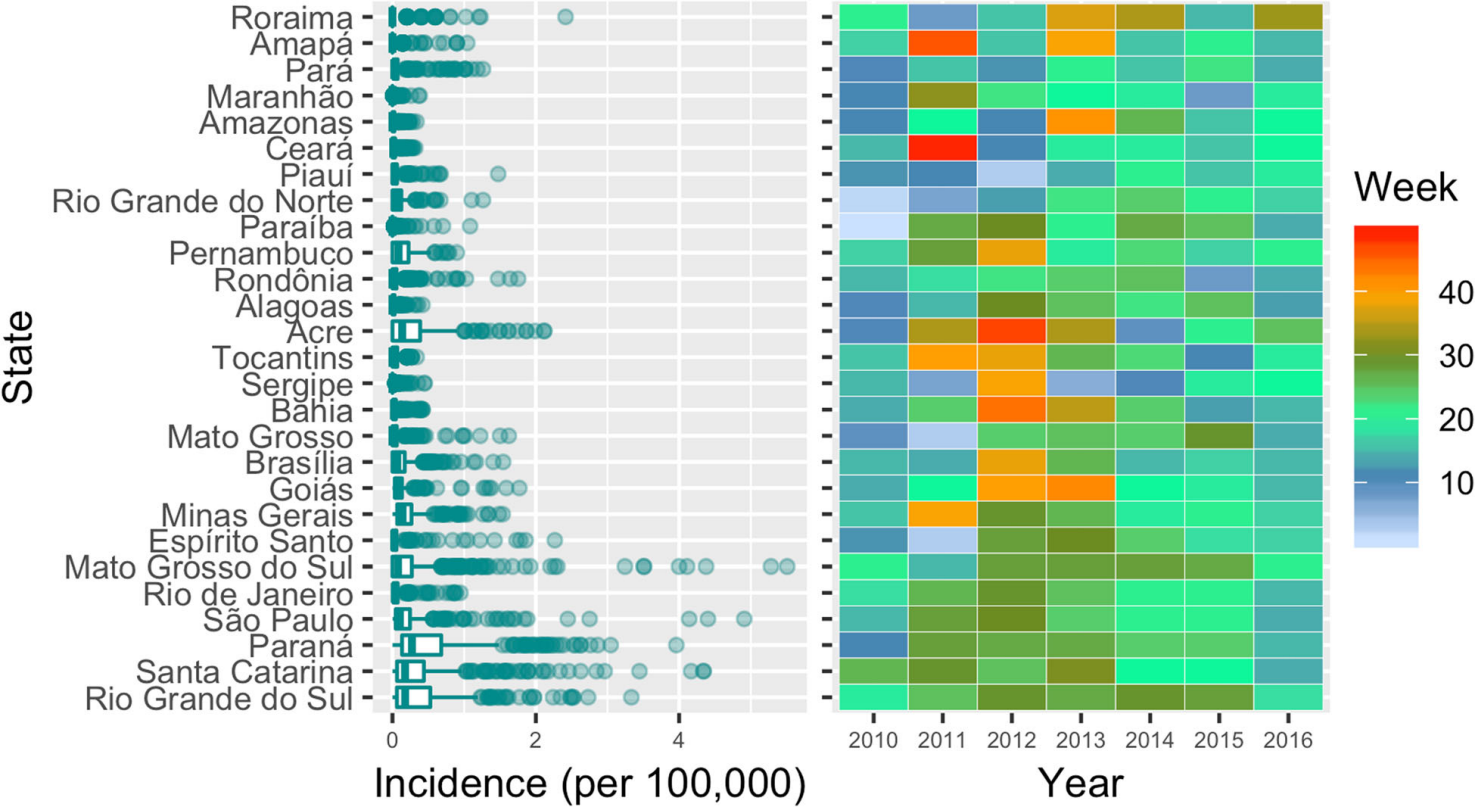
Distribution of incidence per 100,000 inhabitants (on the left), and peak-week by year (on the right) for all Brazilian states and FD, ordered by capital latitude, north on top

For a first overview of the data, we conducted an exploratory data analysis before the use of the time series methods. Figure 1 shows the distribution of incidence from all Brazilian states, ordered by latitude, north on top.

The boxplots on Fig. 1 highlight the overall tendency of higher SARI incidence in the higher latitudes of the south region. The state with the largest median incidence is Parańa, followed by Rio Grande do Sul and Santa Catarina. These are the southernmost states of the country, the only ones: (i) lying entirely below the Tropic of Capricorn, and (ii) with subtropical climate [23].

But this latitude effect alone does not capture the true dynamics of the disease. Qualitatively, the heat map on Fig. 1 demonstrates a clear trend towards influenza infection in winter months of lower latitude states. It shows the yearly evolution of the peak week of activity for each state. There, blue and red colors are around summertime, whereas the greens are close to the winter. It is only in the bottom part of the graph (South region) that we see a relation between the winter season and the SARI peak activity over the years.

### Wavelet analysis

**Fig. 2:**
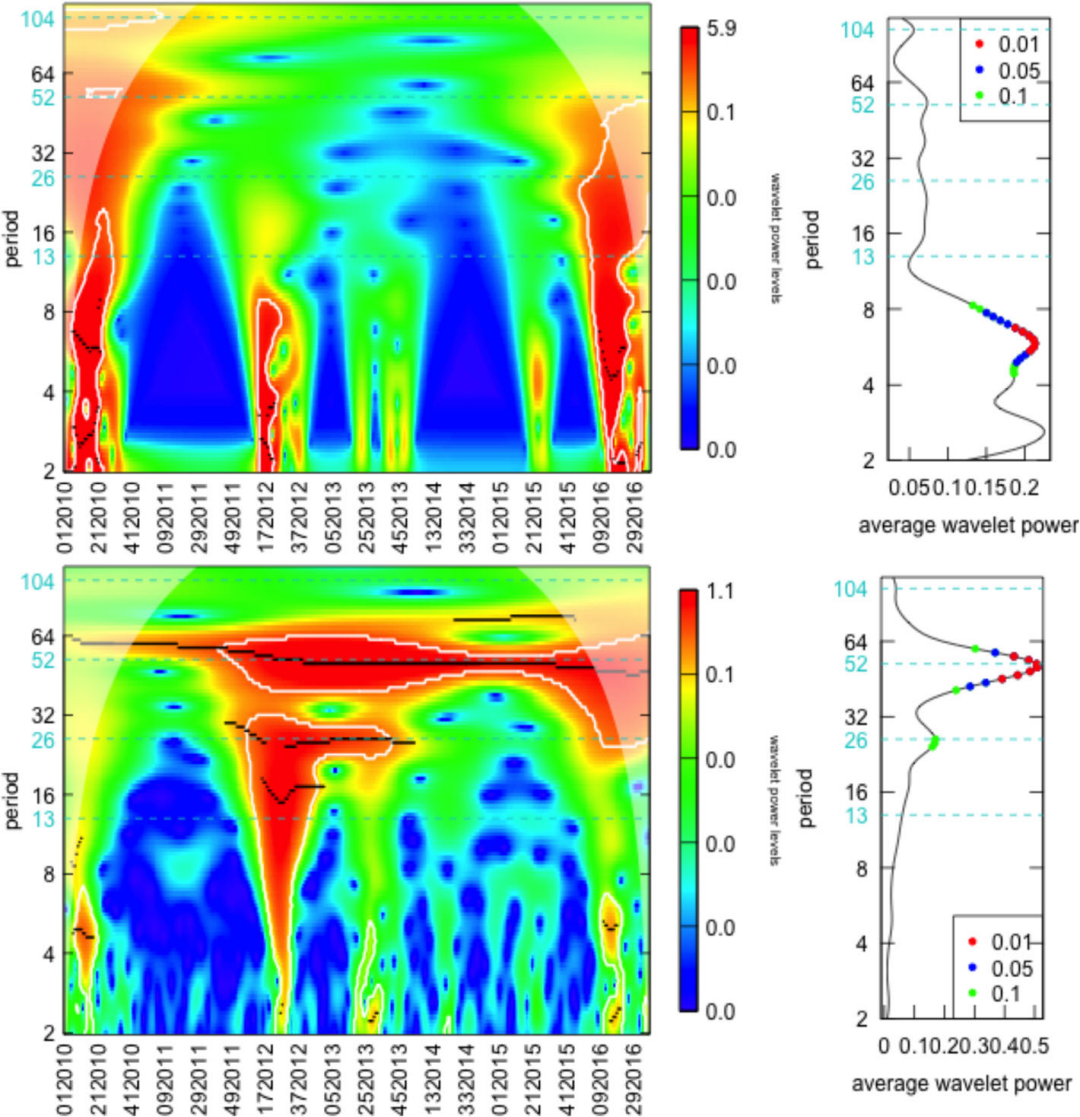
On the left, the wavelet power spectrum where: (i) values increase from blue to red, (ii) white contour lines indicates the 95% confidence interval and (iii) shaded regions on both ends delimiting the cone of influence. On the right, the graphic represents the average wavelet power over time, where dots indicate significant periods at different significance levels. In the top line, the results of Amapá, a northern state and; in the bottom, the Paraná wavelet results, in the south of the country

The wavelet power spectrum is shown in Fig. 2. On top is Amapá, a northern state crossed by the equator line, and on bottom is Paraná, a state in the South region. The seasonal signatures could hardly be more distinct. The wavelet power spectrum of Amapá shows no annual periodic pattern.

The average wavelet power corroborates this conclusion displaying no significant seasonality in mean at the 52 period. The average power around the 52 week period is approximated zero. Just higher frequencies (lower periods) have some explanatory power and are significant in mean.

In Paraná, the temporal pattern displays a consistent seasonal signal for the 52 week period during the whole study period. There is some variability in strength over time, with larger *p*-values at the beginning of the sample. Even so, it remains below the 1% value in every period and therefore it is also significant in mean at the 1% level. The figure also shows semi-annual seasonal phenomena in the middle of the sample, barely significant in mean at the 10% level. The null hypothesis is the best fit ARIMA model, and the alternative is the wavelet decomposition. Graphs for all other states are in the Additional file 1.

**Fig. 3:**
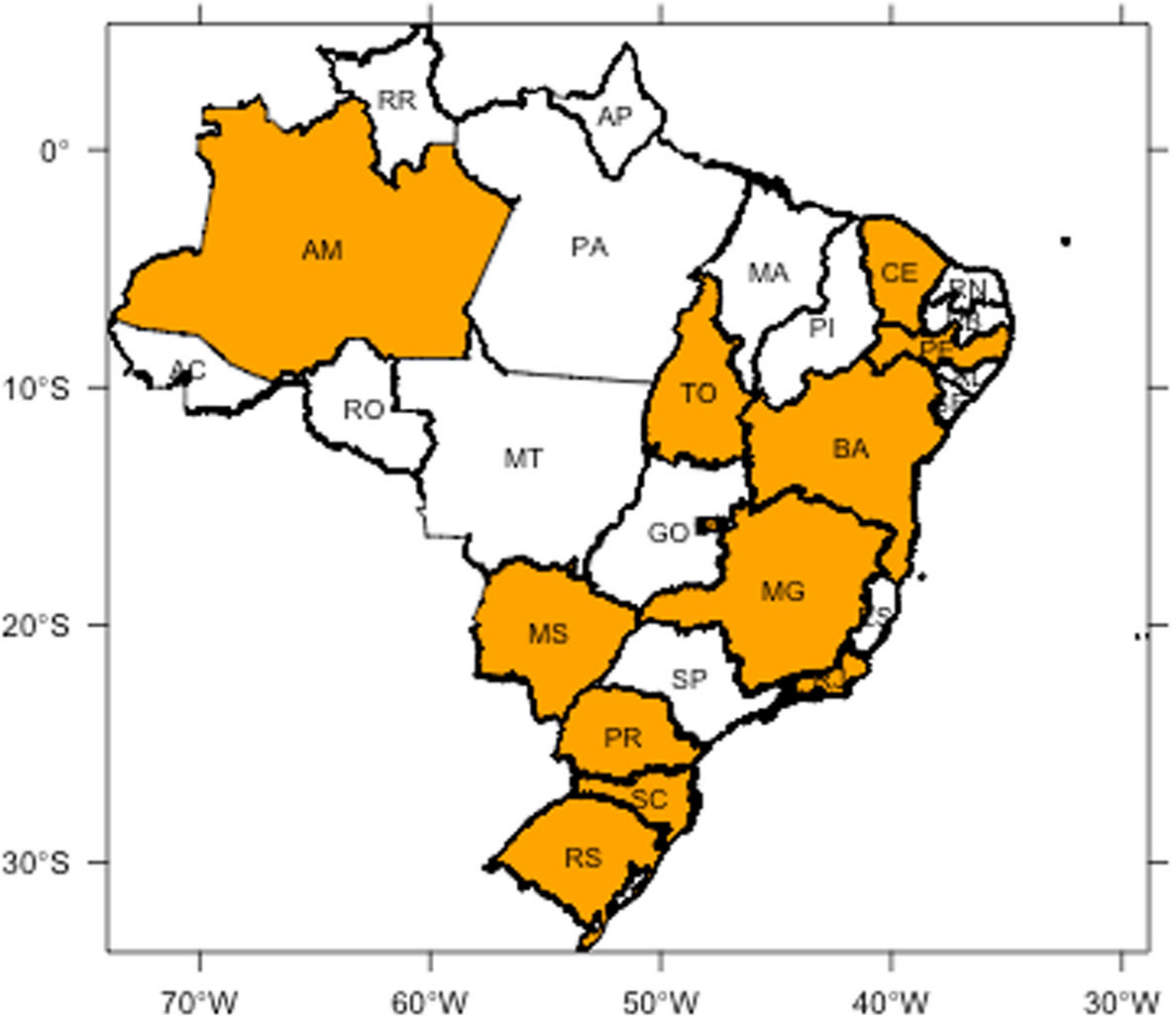
States classified as annual-seasonal (in orange)

The wavelet method did not detect a significant annual pattern in all 27 states: only twelve states, or 44% exhibit some periodic behavior over the 7 years SARI time series. In Fig. 3 we highlighted in orange the states with annual periodicity, significant at the 5% level. They seem to cluster on the coastline, with Amazonas as the only North region exception.

### Circular statistics

**Fig. 4:**
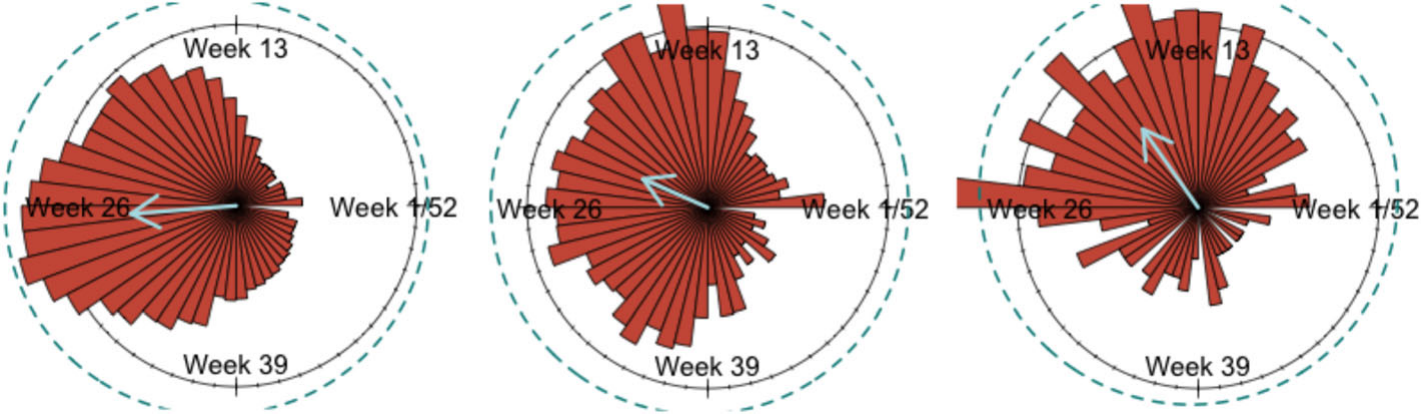
Rose diagram for SARI incidence in Rio Grande do Sul, Brasília (Federal District) and Amazonas (from left to right). The von Mises adjusted probability density function in dashed blue ellipse. The year progresses counterclockwise

**Fig. 5:**
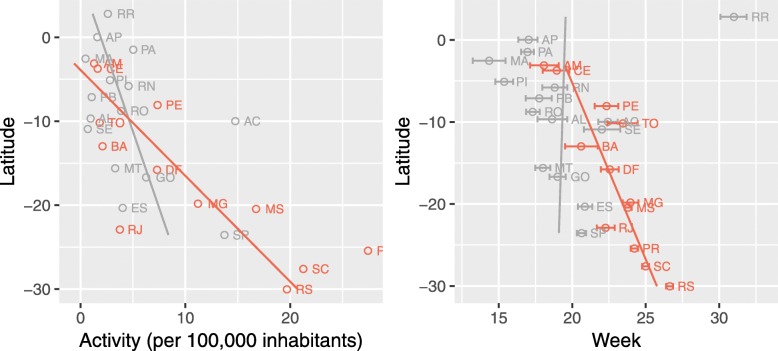
Annual mean activity (left), mean timing of activity/center of gravity (right) by latitude. Results for states with annual seasonality in coral, and non seasonal in gray

Once we have delimited the states showing evidence of annual seasonality using the wavelet decomposition, we applied the second stage of the hierarchical analysis and calculated descriptive circular statistics for the seasonal states. The primary graphical tool to visualize the data circularly is the Rose Diagram. We produced the rose diagrams, their respective mean activity timing or center of gravity, and the average activity for all Brazilian states within the seven-year dataset. In Fig. 4 we present three of them, chosen to represent different latitudes, South on the left. A change in the direction of the mean vector is visible: as we move South, the gravity center goes towards later weeks. The result for all states is presented in Fig. 5.

For the twelve annual seasonal states, we performed the Rayleigh’s test of uniformity. Within the von Mises model, the test is performed on the estimated *κ* concentration parameter. A zero value points to a uniform distribution on the circle. In all these states we rejected the null hypothesis of a uniform distribution.

Figure 5 displays the circular statistics: average year activity and gravity center by state capitals latitude (north on top). The orange colored are the states that exhibit annual seasonal behavior. Confidence intervals were calculated by circular bootstrap methods described in [33]. We initially included the states with no annual seasonality for illustrative purposes only, but interestingly the resultant graph displays more than we expected.

The annual mean activity is shown in Fig. 5 displays the expected behavior, with influenza getting more active as we move south, towards a more temperate climate. The Northern states have a year mean activity 75% lower than the Southern ones, showing that the intensity of SARI incidence across the country varies considerably. Additionally, the graph reveals that the relation between activity and latitude is more pronounced in the seasonal states.

For the mean timing of activity (gravity center), we find a quite interesting result (Fig. 5): influenza comes as a wave starting in the North, with a delay in the peak activity as we move south, but only within the seasonal states. The wave was not found in non-seasonal states.

It is also noticeable that the southern states have a smaller (95%) confidence interval for the centers of gravity. There, the SARI incidence is more concentrated around the mean, denoting a more precise beginning and end of the epidemic through the years.

In Northern states like Ceará and Amazonas, the critical activity peaks are around week 18-19, while in the Southern states of Santa Catarina and Rio Grande do Sul it occurs around week 25-27.

The statistical analyses for all states can be found in the Additional file 1.

## Discussion

In this study, we analyzed a 7-year weekly dataset of Brazilian state-level SARI time-series incidence from 2010 until 2016. The analyses have two primary goals: (i) inferring the existence of annual seasonality pattern for each state, and (ii) describing how summary statistics related to the seasonal activity can vary in a country with an extensive range of latitudes.

It is the first study (known to us) to use Brazilian SARI incidence to infer about influenza seasonality and evaluate this across all Brazilian states.

Influenza within temperate climate zones is known for their well defined seasonal pattern. This pattern is similar to those found in the Brazilian Southern (subtropical) states, the ones that are most affected by the disease, as shown in Fig. 5 and also by [38, 39].

Some authors have shown the existence of a latitude relationship with the spread of influenza [4, 10, 15–17, 19, 20, 22]. Most of them associate it to climatic variables like temperature, precipitation, humidity or hours of sunshine [5, 15, 17, 18, 20, 40].

In particular, Alonso et al. [10] conducted an important study on the pat- tern of mortality by influenza and pneumonia in Brazil. Their main finding was a *wave from the north*, a pattern of disease peak activity starting earlier in the North of the country in May, with delayed peak activity in the South, in July.

Alonso et al’s study describes a well-behaved pattern for the aggregate Brazilian data: in their case, monthly deaths from 1979 to 2001, whereas our dataset comprises weekly SARI hospitalization/deaths cases from 2010 to 2016. A similar pattern is observed in only the southern states in the data presented here. The behavior of the time series for other states do not exhibit this trend. The behavior of the time series for other states tend to be far more erratic in nature, and seasonal behavior is found only in some cases.

Their approach did not benefit from the modern wavelet technique. Hence, no statistical analysis for reassuring that a given state-level series is seasonal is conducted. Also, for some states the confidence intervals reported are quite large: for the Rondônia, Acre and Mato Grosso the reported 95% confidence interval have lengths of 10.5, 9.6 and 11.4 months. In our dataset, these states exhibit no seasonal behavior.

According to our results, we propose a different hypothesis for the traveling wave: it does not start in the Amazon but instead in the Northeast, moving south along the coast. Our proposition is based is several pieces of evidence.

First, the seasonal signature in the North region as a whole is weak. From the six states of the region only one, the state of Amazonas was found to be seasonal. Additionally, seasonality trends are not as robust for this region. An inspection of the Amazonas power spectrum shows a seasonal behavior only in the second half of the sample. Hence, should more stringent criteria for seasonality be adopted, Amazonas would no longer be considered seasonal. To confirm the lack of seasonality for Amazonas, an inspection of the rose diagrams on Fig. 4 shows very complex phenomena, with clear multimodality. Events in March and July are as important as the seasonal peak in May. These suggest that perhaps the seasonal behavior detected in the state of Amazonas is accidental, a type I statistical error.

Alonso et al were puzzled that the wave started from extremely sparsely populated areas in the Amazon basin where roads are limited. Therefore, the wave would have no conceivable connection with the movement of people, since travel by air would likely move the disease directly to the most populated areas in the Southeast region.

Amongst the states that exhibit seasonal behavior, Amazonas peaks first in week 18, closely followed by Ceará (week 19), in the Northeast. Ceará is a far more likely starting point for the influenza season. Its capital, Fortaleza, has around 4 million inhabitants in the metropolitan area, the largest in the Northeast region. A possible explanation for why the wave starts in Ceará is the rainy season spanning from March to May, peaking around week 15. It is true that influenza is associated with cold and dry climate in temperate regions, but little is known in places where it never gets cold. In Fortaleza, the monthly maximum temperature oscillates between 29.1*C*^*°*^ and 30.4*C*^*°*^ year round. In fact, locals call this rainy season as the *winter* season, the only time of the year with a somewhat cooler sensation. The rainy season, therefore, may be the trigger for the disease.

Another piece of evidence suggesting that the wave starts in Ceará is the better mechanism of how it may move southbound. Viboud et al. [41] found that movement of people to and from work was a key determinant of regional disease spread. According to their result, a wave starting in Ceará is far easier to explain. The reason is that some of the main roads of the country are located alongside the coastline from Ceará to Rio Grande do Sul, so the movement of people and cargo there is constant and intense.

Regarding the five administrative regions in Brazil, two seem disconnected from this wave from the Northeast: the North and the Midwest regions. They possess 3.07 and 11.15 km of paved roads per million square km of territory, respectively. The numbers for the Northeast, Southeast and South regions (comprising most of the states in the wave) are 24.28, 37.00 and 31.23, respectively. For a rough comparison, the USA corresponding number is 450, while its GDP is ten times that of Brazil. [42]. So, it is possible that their isolation impacts the spread of influenza in this area.

Finally, we must mention that the state of São Paulo also exhibits a deviation from the expected pattern. It is Brazil’s most populated state, contributing to 1/3 of its GDP, where a severe influenza epidemic occurred in 2016. Its peak was in week 14, whereas the gravity center from 2010 to 2015, that is, excluding 2016, was the 21.03 week. The exercise of excluding the 2016 cases was conducted and resulted in São Paulo being seasonal. We, however, maintained its non-seasonal status in the study.

Climate, location and road infrastructure seems to play a complex role in the influenza seasonality and the spread dynamics. We aim not at ex- plaining all these factors, but rather at obtaining a practical result: a good characterization of the wave and consequent identification of the epidemic peaks across different latitudes. This characterization is rather important to improve the vaccine logistics in a continental country with limited resources. Today, Brazil follows a common vaccination calendar [11].

We restricted ourselves to a single definition of dynamic seasonality, using time averages. The flexibility of the wavelet analysis allows many other possibilities. The power spectrum offers a *p*-value for each observation in each frequency. For instance, Paireau et al. [43], consider the time series to be seasonal using a criterium of three consecutive years of significant *p*-values in a given frequency.

Another way our analysis could be further enriched is through the use of multimodal models in the circular context. We tested for a uniform distribution against a van Mises which is unimodal. A nested test for an increasing number of von Mises in a mixture model is feasible, but have not been implemented.

Little is known on why the seasonality of influenza varies with latitude [44]. Our results can be used to generate hypotheses about factors potentially involved in this unknown relationship. No single environmental factor has been shown to be the driver: in India, Chadha et al. [22] mention the rainfall; in China, temperature, hours of sunshine, and maximum rainfall [15] appears to have important relationships to the disease spread.

Further studies will be necessary to try to uncover the causes of this relationship. Climatic factors are probably essential drivers in the relationship between latitude and influenza spread, but why states within the same climatic zone show such a diverse pattern (no seasonal *versus* seasonal) is still a matter for further study.

## Conclusion

We conducted a dynamic time-series analysis to advance the understanding of seasonal influenza dynamics in tropical lands. In Brazil, only 12 of the 27 states were found to have annual-seasonal influenza activity. Among them, we identified that the peak activity starts in Ceará, the largest city of the Northeast region in 19^th^ week, traveling southbound by areas covered by roads with constant movement of people. The peak in the Rio Grande do Sul, the southernmost state of Brazil occurs in the 27^th^ week. Our results can be used to help the country vaccine logistics, identifying states where an earlier schedule is desirable, and others where some postponement will not harm. The span of the peak activity is more than two months apart, so the benefits of a noncommon vaccination calendar are not trivial.

## Additional file

Additional file 1: All statistical analyses associated with this article can be found online at http://www.worldwidewavelets.com/. (DOCX 12 kb)

## Abbreviations

SARI: Syndrome of Acute Respiratory Disease
SINAN: Notifiable diseases information system
IBGE: Brazilian Institute of Geography and Statistics
