# Correction to: Seasonal dynamics of influenza in Brazil: the latitude effect

**DOI:** 10.1186/s12879-019-3739-3

**Published:** 2019-03-04

**Authors:** Alexandra Almeida, Cláudia Codeço, Paula M. Luz

**Affiliations:** 1 Escola Nacional de Saúde Pública, FIOCRUZ, Rio de Janeiro, Brazil; 2 Programa de Computação Científica, FIOCRUZ, Rio de Janeiro, Brasil; 3 Instituto Nacional de Infectologia Evandro Chagas, FIOCRUZ, Rio de Janeiro, Brasil

**Correction to: BMC Infectious Diseases (2018) 18:695**

**https://doi.org/10.1186/s12879-018-3484-z**

Following publication of the original article [1], the author reported her name has been erroneously spelled as Paula Luz.

The publisher apologizes for any inconvenience caused by this error.
