# Supplementary material for: Seasonal dynamics of influenza in Brazil: the latitude effect
Source: BMC Infect Dis. 2018 Dec 27;18:695. doi: 10.1186/s12879-018-3484-z (PMC6307116; doi:10.1186/s12879-018-3484-z)
Supplement: Supplementary file 1 — All statistical analyses associated with this article can be found online at http://www.worldwidewavelets.com/. (DOCX 12 kb) [file 12879_2018_3484_MOESM1_ESM.docx]

http://www.worldwidewavelets.com/
